# The impact of inequalities and health expenditure on mortality due to oral and oropharyngeal cancer in Brazil

**DOI:** 10.1038/s41598-021-92207-x

**Published:** 2021-06-18

**Authors:** Amanda Ramos da Cunha, Alessandro Bigoni, José Leopoldo Ferreira Antunes, Fernando Neves Hugo

**Affiliations:** 1 Faculty of Dentistry, Federal University of Rio Grande do Sul, 2492 Ramiro Barcelos St, Porto Alegre, RS 90035-003 Brazil; 2 School of Public Health, University of São Paulo, 715 Doutor Arnaldo Ave, São Paulo, SP 01246-904 Brazil

**Keywords:** Cancer epidemiology, Oral cancer

## Abstract

This study aims to assess the magnitude and trend of mortality rates due to oral (OC) and oropharyngeal cancer (OPC) in the 133 Intermediate Geographic Regions (IGR) of Brazil between 1996 and 2018 and to analyze its association with sociodemographic variables and provision of health services. It also aims to compare the trend of mortality from neoplasms that have been reported as associated with HPV (OPC) with the trend of neoplasms that have been reported as not associated with HPV (OC). We obtained mortality data from the Mortality Information System in Brazil and analyzed the trends using the Prais-Winsten method. Then, we assessed the relationship between mortality trends and socioeconomic, health spending, and health services provision variables. The median of the annual percent change of the country’s mortality rates was 0.63% for OC and 0.83% for OPC. Trends in mortality in the IGRs correlated significantly with the Human Development Index and government expenditure on ambulatory health care and hospitalizations. Mortality from both types of cancer decreased in those IGR in which the government spent more on health and in the more socioeconomically developed ones. This study found no epidemiological indication that HPV plays the leading etiological factor in OPC in Brazil.

## Introduction

The incidence and mortality from oral and oropharyngeal cancer (OC and OPC) is a global concern. The International Agency for Research on Cancer (IARC) has estimated more than 500,000 new cases and more than 250,000 deaths from these cancer subtypes for 2018^1^. In 2017, oral and pharyngeal neoplasms accounted for 4% of cancer deaths worldwide, according to estimates from the Global Burden of Disease Study (GBD)^2^. In Brazil, the National Cancer Institute (INCA) estimated for 2020 an incidence rate of OC of 9.25/100,000 inhabitants among men, being the 5th most common type of cancer in the male population^3^.

The literature reports a negative association between the risk of OC and OPC and socio-economic conditions. Conway et al.^4^ evaluated the effect of individuals' income and education on the risk of developing head and neck cancer. The authors identified that the risk of developing this type of cancer was twice as high at the lowest levels of income and education. Concerning OC, similar results were obtained^5^. Access to health services also seems to influence the pattern of incidence and, especially, of mortality from OC and OPC. Survival rates are associated with the tumor’s stage at diagnosis and seem to be related to the availability and quality of the treatment offered^6–8^. Considering Brazil, which is the fifth-largest country in the world and presents enormous social inequality^9–11^, the lack of knowledge about the dynamics of the disease and their relationship with the different characteristics of the territory—such as the availability of health services or the level of socio-economic development—are obstacles to the planning of actions tailored to the needs of the population.

The description of the magnitude and trend of cancer incidence and mortality rates is crucial for understanding its evolution in the population context. There is a significant variation in the distribution and temporal trends of oral and oropharyngeal neoplasms worldwide, explained by the different exposure patterns to risk factors. Analyzing the trend in the incidence of these diseases, Chaturvedi et al.^12^ verified a variation in the etiological profile of OC and OPC between high and low/middle-income countries. The authors identified an increasing pattern in OPC incidence, contrasting with a decreasing (or stable) trend in OC incidence in high-income countries. This difference was attributed to the growing role of the HPV virus in oropharyngeal cancer's etiology—which is currently considered the leading risk factor for OPC in some countries, like the USA^13^-, concomitant with the reduction in the prevalence of tobacco consumption in these countries, the most classic risk factor for OC^14^. Considering mortality, HPV-related neoplasms have a better prognosis (are less lethal and favor greater survival) than those not related to HPV^15,16^. Due to this difference, the analysis of mortality trends may provide clues about the etiological patterns of these diseases. Concerning low and middle-income countries, tobacco still seems to be the leading risk factor for both types of cancer^12^, but this assumption still need support from further evidence. Like Brazil, most countries do not have cancer databases that inform, for example, the HPV serology of tumors. Knowing the epidemiological behavior of these diseases, from a perspective that can elucidate aspects related to its etiology—i.e., comparing OC with OPC trends-, is necessary for the timely prevention of this condition, as it allows identifying changes in its patterns.

In 2017, the Brazilian Institute of Geography and Statistics (IBGE) divided the Brazilian territory into 133 Intermediate Geographic Regions (IGR). The IGR corresponds to an intermediate geographical stratification between states and cities. These territorial delimitations always include large urban centers as a reference for urban functions of greater complexity to the neighboring towns^17^. Among other characteristics, IGR tends to cover the path through the health system that a patient, resident of these, goes through; larger cities act as a reference (of different natures, including health) for smaller cities. If a cancer patient lives in a small city without a hospital, he/she is likely to be referred for hospital treatment in a more structured city close to his local residence, which serves as a reference for more complex urban functions for that region. This characteristic makes IGR an opportune territorial division to study the determinants of mortality from OC and OPC, identifying which factors are associated with more favorable regional outcomes. Nevertheless, to this date, no study examined if mortality due to OPC and OC varies by IGR.

The present study aims to assess the magnitude and trend of mortality rates due to OC and OPC in the IGR of Brazil between 1996 and 2018 and to analyze its association with sociodemographic factors and variables assessing provision and use of health services. Additionally, it aims to compare the trend of mortality from neoplasms that have been reported as associated with HPV (OPC) with the trend of mortality from neoplasms that have been reported as not associated with HPV (OC)^18^—which is a methodological approach established in the literature—^19,20^, respecting current knowledge, that considers these two diseases as distinct clinical and epidemiological entities^19^. By detailing this analysis by IGR and understanding how these rates behave in different profile territories, we expect to counterbalance the over-generalization present in most existing studies.

## Methods

This ecological study used data from the Mortality Information System (SIM, for the acronym in Portuguese) of Brazil from 1996 to 2018. The study period begins in 1996, when the SIM adopted the tenth revision of the International Classification of Diseases (ICD-10)^21^ and ends in 2018, which is the most recent year with available data in the SIM at time of the present study. The database encompasses all deaths whose underlying cause was oral cancer (C02.0–C02.3, C02.8, C02.9, C03–C06) and oropharyngeal cancer (C01, C02.4, C09, C10 and C14.2). The selection of which ICD-10 codes make up each type of cancer was made based on the proposition of Chaturvedi et al.^22^; it is described in Supplementary Chart S1. As proposed by different previous studies^19,20^, this classification aimed to compare the trend of mortality from neoplasms that have been reported as associated with HPV (OPC) with the trend of mortality from neoplasms that have been reported as not associated with HPV (OC). We also collected information about sex, age, and residence local of each case.

The Regional Office of the United Nations Development Program informed the Human Development Index (HDI) for each Brazilian town in 2010 (the most recent information available). The Information System on Primary Health Care maintained by Brazil's Ministry of Health provided the number of Family Health Teams (FHT) in each town, thus allowing calculating the coverage of the Family Health Strategy (FHS). As this number is informed for each month, we used the averaged 2018 coverage data. The Ambulatory Information System (SIA/SUS) and the Hospital Information System (SIH/SUS) provided 2018-related data on government expenses related to outpatient health care and hospitalizations, respectively, in terms of Brazilian Reals, the official currency in the country. The official demographic agency in the country (the Brazilian Institute of Geography and Statistics—IBGE) provided population data for each town in 2000 and 2010 (census years), and intercensal estimates for the remaining years.

The 5570 Brazilian towns were gathered into 133 Intermediate Geographic Regions (IGR) of the country. The IBGE created this territorial division as an intermediary administrative hierarchical level encompassing nearby towns within the same state^17^. Death rates due to oral and oropharyngeal cancer were calculated per 100,000 inhabitants for each IGR. The rates were adjusted by sex and age group (5-year intervals), using the direct method and the population distribution proposed by the World Health Organization^23^—the rates were also adjusted by the Brazilian population in 2010; however, the results were remarkably similar for the two adjustments. Therefore, to increase the comparability of this study's results, the standardization by the population proposed by WHO was prioritized.

Deaths with absent information on sex, age, or local residence were redistributed based on the proportion of deaths with existing information. Deaths by ill-defined and unspecified causes were also redistributed proportionally, according to the method of reclassifying garbage codes proposed by the Global Burden of Disease Study 2010^24^—we provided more details of how we applied this correction method in the “Supplementary material” (Section “Redistribution of ill-defined deaths” and Supplementary Chart S2).

The outcome on cancer mortality and all covariates were calculated for each IGR. The HDI of each IGR was obtained by a weighted average of the index in each town, using the number of inhabitants of the town as the weighting factor. The FHS coverage was calculated using an analogous scheme. The number of FHT was divided by the population in the respective year, multiplied by 3000, and, finally, multiplied by 100 to obtain the percentual coverage for each town. According to the Ministry of Health, this number (3000) is the ideal number of inhabitants for each FHT^25^. Government expenses related to outpatient procedures and hospitalizations per capita were calculated directly for each IGR: the total annual amount, in Reals, was divided by the population in the same year. The Supplementary Chart S3 summarizes information about exposure variables.

For the trend analysis of mortality between 1996 and 2018, we applied a generalized linear regression model using the Prais-Winsten method, in which the dependent variable was the log-transformed death rate, and the independent variable was the year of death. The Prais-Winsten method allows assessing the trend with correction for first-order autocorrelation. In addition to improving the goodness of fit of the models^26,27^, the log transformation allows calculating the annual percent change (APC). The assessment APC and its respective 95% confidence interval (CI95%) used the formula recommended by Antunes and Waldman^28^:

$$\begin{aligned} & APC = \left( { - 1 + 10^{{b1}} } \right)*100\% \\ & CI_{{95\% }} ^{{lower}} = \left( { - 1 + 10^{{b1lower}} } \right)*100\% \\ & CI_{{95\% }} ^{{upper}} = \left( { - 1 + 10^{{b1upper}} } \right)*100\% \\ \end{aligned}$$

In which *b1* is the regression coefficient, and *b1lower*/*b1upper* are the limits of its CI_95%_. This procedure allows to classify the rates as ascending (APC and CI_95%_ positive), declining (APC and CI_95%_ negative), or stationary (CI_95%_ that includes the zero). The APCs were assessed for each IGR and thus aggregated by the Brazilian macro-regions: North, Northeast, Southeast, South, and Midwest.

We used Pearson's correlation to analyze and quantify the association between the APCs and the covariates HDI, FHS coverage, and public expenditure on health. The IGRs were classified into quartiles for each covariate—the first quartile refers to the IGRs that concentrated the 25% lower HDI values of the sample (i.e., the less developed ones) and lower government spending; the fourth refers to the IGRs with the 25% highest values. Finally, we graphically compared the magnitude of OC and OPC mortality in the initial years of the series (average rates for 1996–1998) with the final years (average rates for 2016–2018) by IGR. All analyses, including the maps' creation, were performed using the Stata 14.0 software. The Brazilian Institute of Geography and Statistics website (https://www.ibge.gov.br/geociencias/downloads-geociencias.html) was the source of the shapefiles that contained the cartographic basis for creating the maps.

To present some results, we aggregate the IGRs results in their respective macro-regions (North, Northeast, Southeast, South, and Midwest). The North and Northeast regions are the poorest of Brazil in terms of Gross Domestic Product (GDP) per capita (respectively, 17,213.30 and 12,954.80 Brazilian Reals) and HDI (0.667 and 0.663)—for the Southeast, these metrics are 34,789.78 and 0.766. While in the Southeast, South, and Midwest, less than 3.5% of the population lives in extreme poverty, this percentage in the North and Northeast is 11.4% and 13.7%, respectively^29,30^”.

## Results

From 1996 to 2018, 73,563 people died from OC, and 63,970 died from OPC in Brazil. The median of the APC of the country’s mortality rates, considering the trend of mortality in the 133 IGRs, was 0.63% for OC and 0.83% for OPC. In general, median APCs were higher in the North and Northeast for both types of cancer (Table 1).

**Table 1:** Annual percent change of oral and oropharyngeal cancer mortality: median and interquartile range (IQR) in Brazilian intermediate geographic regions, by sex, and macro-region.

	Sex	N (n. 22)	NE (n. 42)	SE (n. 33)	S (n.21)	MW (n. 15)	Brazil (n. 133)
		Median (IQR)	Median (IQR)	Median (IQR)	Median (IQR)	Median (IQR)	Median (IQR)
OC	G	0.84 (− 0.33; 1.88)	2.15 (1.36; 2.74)	− 0.03 (− 0.85; 0.84)	− 0.95 (− 1.65; − 0.13)	− 0.16 (− 0.74; 2.00)	0.63 (− 0.83; 2.00)
	F	− 0.93 (− 3.49; 0.43)	0.24 (− 1.10; 1.59)	0.06 (− 1.17; 0.49)	− 0.07 (− 1.23; 2.15)	− 0.76 (− 1.92; 1.98)	− 0.34 (− 1.55; 0.73)
	M	1.76 (− 0.31; 3.30)	2.82 (1.20; 4.22)	0.37 (− 0.95; 1.27)	− 1.08 (− 1.62; − 0.39)	0.88 (− 0.94; 3.86)	0.88 (− 0.72; 2.90)
OPC	G	1.08 (− 0.48; 2.91)	2.66 (1.30; 3.92)	− 0.24 (− 1.13; 1.72)	− 0.17 (− 0.83; 0.32)	0.43 (0.04; 1.00)	0.83 (− 0.61; 2.62)
	F	− 1.11 (− 4.18; 1.71)	0.07 (− 1.14; 1.61)	− 0.95 (− 1.65; − 0.13)	− 1.46 (− 2.53; 0.25)	− 1.32 (− 2.73; − 0.76)	− 0.80 (− 1.91; 0.95)
	M	1.61 (− 0.15; 4.86)	3.29 (1.63; 4.90)	− 0.07 (− 1.23; 2.15)	− 0.20 (− 0.69; 0.37)	0.87 (0.50; 1.99)	1.43 (− 0.32; 3.48)

Brazil, 1996–2018.

*OC* oral cancer, *OPC* oropharyngeal cancer, *G* general, *F* female, *M* male, *N* North, *NE* Northeast, *SE* Southeast, *MW* Midwest.

**Figure 1:**
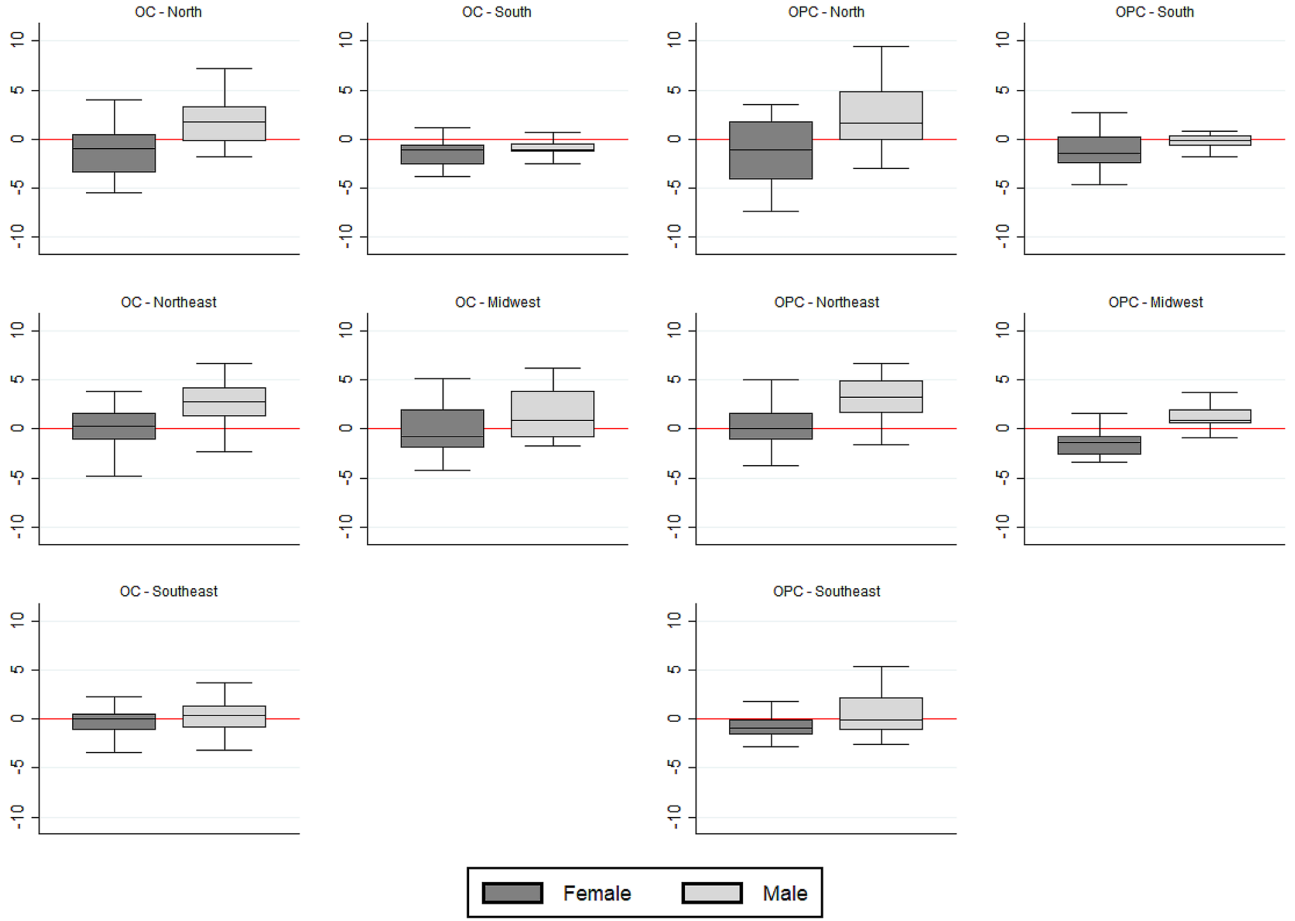
Annual percent change of oral (OC) and oropharyngeal cancer (OPC) mortality: variation across the intermediate geographic regions (IGR), by sex, and macro-region. Brazil, 1996–2018.

The Northeast had the steeper increase of OC death rates (median APC = 2.15%); whereas the South had the lowest median APC (− 0.95%) (Table 1). Oral cancer mortality was stationary in most IGRs. The Northeast had the highest proportion of IGRs with increasing trends (59.5%), while no IGR had an increasing trend in the South (Supplementary Tables S1–S5). When comparing the sexes, the median APC was higher for men than for women in all regions, except for the South. The larger discrepancy between the sexes occurred in the North and the lower in the Southeast (Fig. 1).

Regarding trends of OPC mortality, the biggest median APC occurred in the Northeast (2.66%), whereas the lowest was in the Southeast (− 0.24%) (Table 1). Oropharyngeal cancer mortality was also stationary in most IGRs of the country. The Northeast had the greatest number of IGRs with an increasing trend (57.1%), and no IGR in that region had a decreasing tendency. The Southeast had the highest proportion of IGRs with decreasing trends (24.2%) (Supplementary Tables S1–S5). When comparing the sexes, the median APC was higher in men than in women in all regions (Fig. 1). Tables S1–S5 of the “Supplementary material” present the results of the trend analysis for OC and OPC by IGR.

**Figure 2:**
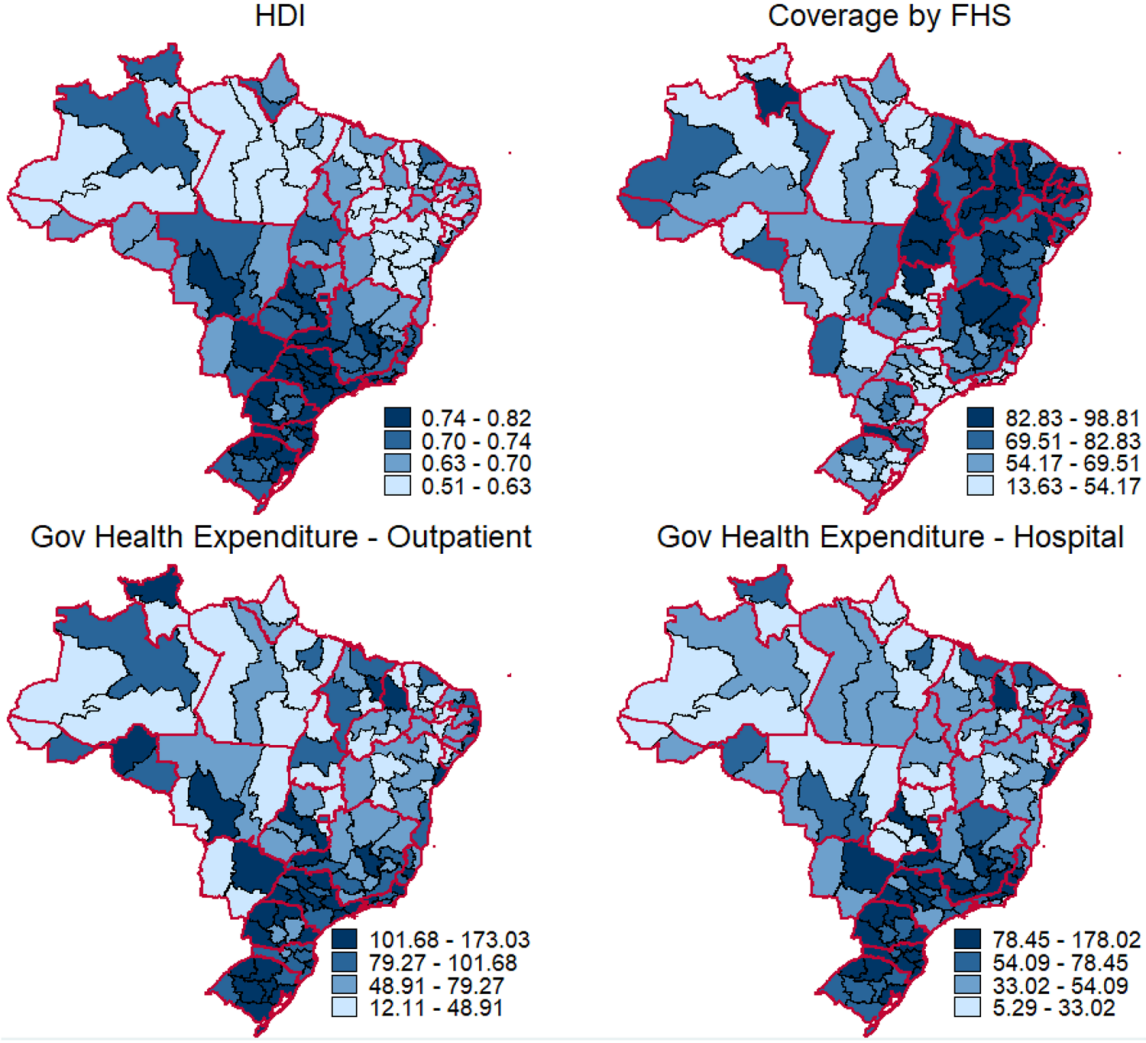
Distribution of covariates in Brazil’s intermediate geographic regions: the Human Development Index (HDI) in 2010; coverage of the Family Health Strategy (FHS) in 2018; and per capita government expenditure in outpatient health care, and hospitalizations, in Brazilians Reals, in 2018. This figure was created by the authors, using Stata 14.0 software and the shapefiles provided by the Brazilian Institute of Geography and Statistics website.

Trends in mortality from OC and OPC in Brazil's IGRs correlated significantly with the HDI, FHS coverage, and government expenditure on ambulatory health care and hospitalizations. IGRs in the South and Southeast regions had higher HDI and per capita government expenditure on outpatient procedures and hospitalizations than the remaining regions. In contrast, IGRs in the Northeast region had higher FHS coverage by (Fig. 2). IGRs in the lowest HDI quartiles tended to present with steeper increasing trends of mortality, i.e., the APC for OC and OPC was inversely associated with the HDI. The correlation between the APC and government expenditure on outpatient procedures and hospitalizations followed the same pattern, except for the mortality of women. The correlation between mortality trends and FHS coverage, however, was positive and statistically significant (Table 2), depicting that the health program (the FHS) provided a higher coverage in the IGRs with higher increase of mortality.

**Table 2:** Trends of oral and oropharyngeal cancer mortality: annual percent change median, by sex, and quartiles of the Human Development Index, coverage by Family Health Strategy, and government expenditure in outpatient health care, and hospitalizations (per capita). Brazil, 1996–2018.

Type of cancer	Sex	1st quartile	2nd quartile	3rd quartile	4th quartile	R
**Human development index**						
Oral	G	2.34	1.37	0.09	− 0.85	− 0.61*
	F	0.46	− 0.32	− 0.82	− 0.54	− 0.23*
	M	3.36	2.08	0.65	− 0.95	− 0.63*
Oropharynx	G	2.90	1.37	0.14	− 1.00	− 0.67*
	F	1.10	− 0.13	− 1.23	− 1.35	− 0.32*
	M	3.72	2.46	0.87	− 0.92	− 0.63*
**Coverage by family health strategy**						
Oral	G	− 0.61	− 0.71	1.14	2.38	0.54*
	F	− 0.56	− 0.54	− 0.36	0.37	0.11
	M	− 0.49	− 0.55	1.83	3.42	0.57*
Oropharynx	G	− 0.61	0.32	1.72	2.74	0.56*
	F	− 1.09	− 0.53	− 1.20	− 0.64	0.12
	M	− 0.48	0.50	2.51	3.79	0.59*
**Gov Health Expenditure (per capita)—outpatient**						
Oral	G	1.69	1.88	− 0.34	− 0.94	− 0.50*
	F	− 0.33	0.06	− 0.20	− 0.64	− 0.10
	M	2.09	2.63	0.09	− 0.94	− 0.54*
Oropharynx	G	2.15	1.35	0.43	− 0.37	− 0.46*
	F	0.03	− 1.05	− 1.30	− 0.85	− 0.02
	M	3.49	2.17	1.01	− 0.20	− 0.50*
**Gov Health Expenditure (per capita)—hospital**						
Oral	G	1.69	1.50	0.21	− 0.69	− 0.50*
	F	0.18	− 0.00	− 0.47	− 0.64	− 0.13
	M	2.23	2.37	0.76	− 0.67	− 0.54*
Oropharynx	G	1.87	1.08	0.42	− 0.24	− 0.41*
	F	− 0.33	− 0.58	− 0.76	− 1.04	− 0.09
	M	2.66	2.17	1.08	− 0.07	− 0.44*

*R* Pearson correlation coefficient, *G* general, *F* female, *M* male.

*p < 0.001.

**Figure 3:**
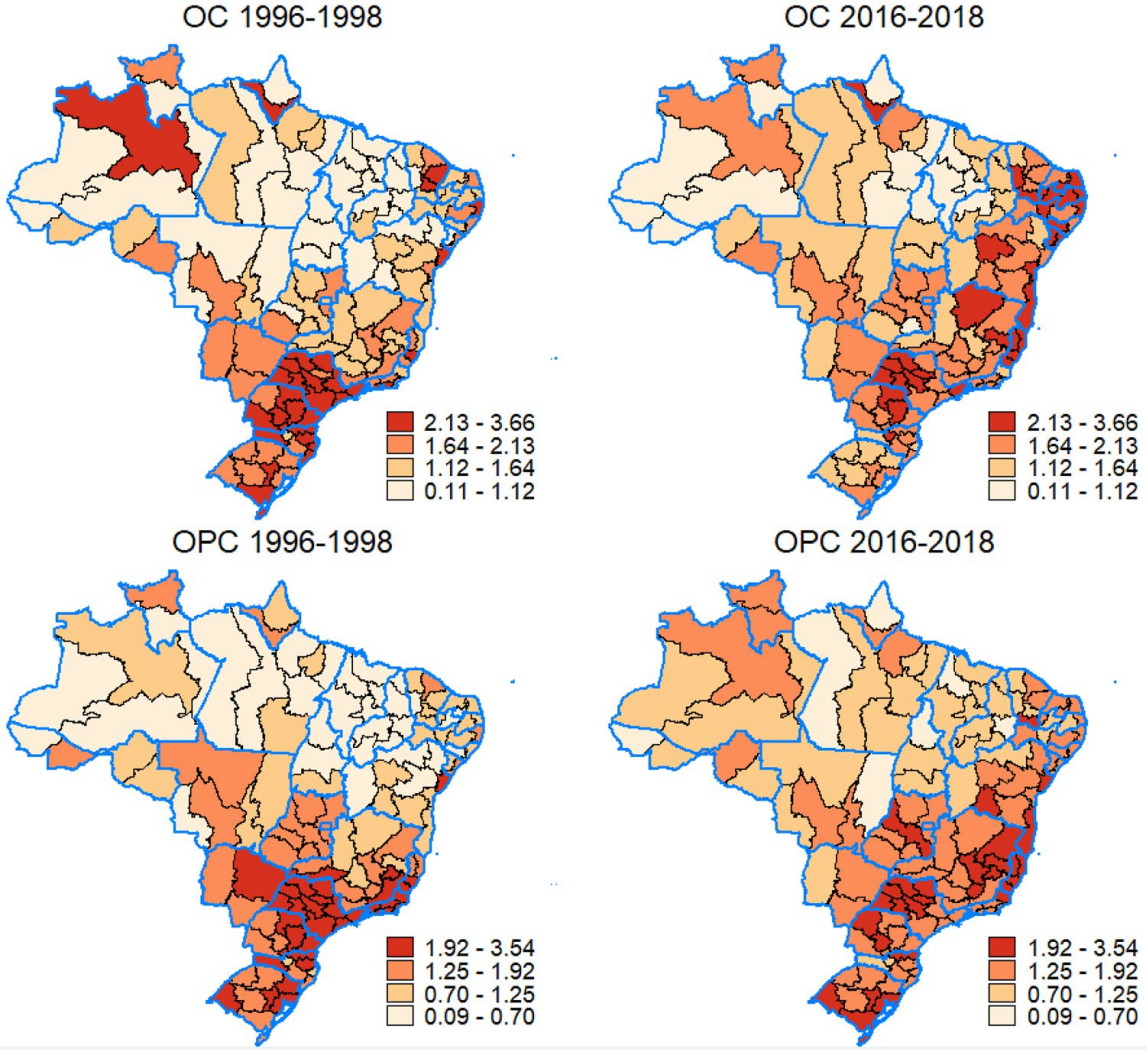
Mortality by oral cancer (OC) and oropharyngeal cancer (OPC): mean death rates at the beginning (1996–1998) and end (2016–2018) of the monitoring, in Brazil’s intermediate geographic regions. This figure was created by the authors, using Stata 14.0 software and the shapefiles provided by the Brazilian Institute of Geography and Statistics website.

When it comes to compare the magnitude of mortality, the richer regions of the country, the Southeast and South, had an overall reduction of death rates from the beginning (1996–1998) to the end (2016–2018) of the study period. However, the opposite occurred in the poorer regions, the North and Northeast, where the rates were higher in the end (Fig. 3).

The median APC of mortality countrywide was higher for OPC than for OC (0.83% and 0.63%, respectively). The same occurred when specifically assessing mortality in men (1.43% and 0.88%). However, this pattern was not homogeneous across the macro-regions. When comparing the two types of cancer—for both sexes—only the Midwest had discrepant trends: the median APC was negative for OC and positive for OPC (Table 1).

## Discussion

This study assessed the trend of mortality from OC and OPC in the last two decades in Brazil and the relationship between these trends and the region's human development, provision of health services, and governmental health expenditures. We found that mortality from both types of cancer decreased in those IGR in which the government spent more on outpatient procedures and hospitalizations and in the more socioeconomically developed ones. These results are the study’s key findings and suggest that government spending in the health sector and a more developed regional socioeconomic structure—represented by HDI—may effectively reduce OC and OPC mortality in the country.

In the analysis of association with health service provision, we found evidence pointing to contrasting scenarios. While governmental expenses with outpatient procedures and hospitalizations correlated negatively with mortality trends for both types of cancer, FHS coverage correlated positively. The results we found for health expenditure per capita have already been shown for other types of cancer^31^, although they have not yet been explored for OC and OPC. Regions that receive more government transfers for hospitalizations are possibly those that have a greater hospital infrastructure and/or deal more extensively with complex therapies, which are more expensive. Our results suggest that this differentiated structure raised more opportunities for treatment, rehabilitation, and cure of OC and OPC cases, which are treatable cancers—namely, cancers that are associated with survival rates that exceed 50% in 5 years^32^. The findings related to outpatient expenditures follow the same logic. IGR that have this level of care more productive may have provided more opportunity for early diagnosis, which is a predictive factor for survival in head and neck cancers^33^.

The findings regarding FHS coverage do not follow the same reasoning. Coverage by these teams represents access to primary health care, which is the main entryway to the Brazilian Unified Health System (Sistema Único de Saúde). A study carried out in Brazil indicated that coverage by Oral Health Teams—which are part of the FHS—was inversely associated with death rates in men from OC and OPC. The authors justified the results explaining that the FHS promoted the expansion of access to health in unassisted areas^34^. Our results indicated that IGRs with more expressive coverage by FHS have greater APC in mortality. We suggest that they are reflecting the pro-equity character of the implementation of the FHS, which prioritizes more vulnerable regions. Studies that evaluated the impact on the population's oral health have ratified this^35^. Thus, coverage by FHS would be acting as a proxy for the IGR's social development in the analysis. Another possibility is that the IGRs with the broader coverages are those that historically had a more insufficient health provision. In this perspective, the FHS compensates for a historical deficiency not because it prioritizes the most deprived regions but because it is the only health care service available—as it is a more financially feasible solution. Our results suggest that the structuring and expansion of health services in Brazil must occur networked and sync with intersectoral policies for social development to compensate for the inequities in illness and death due to OC and OPC.

We identified that the HDI showed a negative association with the mortality trend, for both types of cancer, considering both sexes and each separately: the more developed the IGR, the greater the decrease in mortality due to OC and OPC. The HDI, which summarizes the dimensions of longevity, education, and income, is lower in the North and Northeast. Explaining this association is complex, as there are several dimensions related to the social determinants of health^36^ involved in this relationship. Socioeconomic factors appear to be involved in OC and OPC mortality since exposure to risk factors. A study carried out in Scotland found that, for individuals living in disadvantaged communities, smoking is a mechanism to deal with the stress generated by personal struggles, including economic deficiencies. The obstacles of living in an environment with few opportunities and few resources would intensify these difficulties^37^. Food pattern, inputs and protective or harmful environments^38^, occupational exposures^39^, and access to health services and health information^5^ are all aspects potentially related to OC and OPC and which may be influenced by socioeconomic characteristics. The negative association between mortality and HDI reported here is compatible with previous studies^40–43^.

A substantial number of studies on the incidence of OC and OPC in high-income countries have shown trends of increase in the rates of incidence due to OPC in the last two decades, contrasting with a decreasing or stable trend for OC^12,44–46^. This epidemiological pattern has been associated with the rising role of the Human Papillomavirus (HPV) in the etiology of OPC^47^, which is not observed in the OC. Considering mortality, HPV-related oropharyngeal neoplasms have a better prognosis than those not related to HPV^15,16^. In the present study, the similarity in mortality trends for both types of cancer suggests that the predominant risk factors are common and are operating with similar intensity in both anatomical regions, which is not compatible with the course of HPV. This conclusion fits with results of Anantharaman et al.^48^. In a study of 1420 cases of head and neck cancer, they investigated the percentage of oropharyngeal tumors positive for HPV-16, which was 60% in the USA, 31% in Europe, and only 4% in Brazil. From this perspective, the most classic risk factor (tobacco use) would be operating as a leading etiological factor for OPC incidence and mortality.

We identified higher APCs in mortality rates due to OPC compared to OC in all macro-regions, except for the Southeast. We would expect an opposite situation in a country where HPV is the protagonist of oropharyngeal carcinogenesis. HPV-related head and neck neoplasms are less lethal than non-related ones^15,16^. A recent multicenter study in Europe found almost 50% of reduction in the risk of death in HPV-16 positive cases of OPC when compared to negative ones^49^. Analyzing a cohort of 235 patients in Scotland, Wakeham et al. observed that patients with high-risk HPV positive OPC had 89% less risk of death than the negative ones^50^.

This study used the Brazilian IGR as a unit of analysis. This construct is the product of a new territorial division, which considers the existence of a hub with more complex urban functions for each region. The IGR division respects the concept of network-territory, which reflects the relationship of social subjects with spaces, incorporating their flows and their connections with different territories^17^. Oral cancer and oropharyngeal cancer are severe diseases, which require diagnostic and therapeutic resources of high complexity, usually present in large urban centers. The use of the territorial division in IGR was the strategy that was used to encompass the entire path of the sick individual, without the burden of over-generalization. Presenting findings stratified by IGR can provide an original panorama, which focused on the whole cancer care network as it considers the patient inter-city flows in the health system. This new panorama can provide managers of different government levels (local, from States, Federal) with valuable information for planning effective actions that reach all care levels and prioritize the most vulnerable areas.

The present study found greater APCs—for both OC and OPC—in the IGRs of Northeast and North regions; South and Southeast had the lowest APCs. A Brazilian study that analyzed several types of cancer and used the same historical period found the same APC distribution pattern^31^. These results highlight the need to prioritize the IGRs from Northeast and North in planning cancer prevention, early detection, and control actions: the burden of OC and OPC is increasing in these regions, and the government spends less on health in them than in the others. Also, these regions' population faces more significant socioeconomic problems, which tends to aggravate their already unfavorable epidemiological scenario.

This study’s main limitation is its data source, which is an information system—the Mortality Information System. In the last two decades, death records in Brazil have improved substantially^51^. However, its completeness and quality in identifying the underline cause of death vary between regions of the country. An international comparative study of cancer mortality excluded Brazil due to problems with registering the underline cause of death^52^. To counteract this limitation, we corrected the number of deaths of each IGR using the methodology of the Global Burden of Disease Study 2010^24^. This approach, in addition to considering ill-defined deaths, corrects the “garbage codes”: the deaths registered with codes that do not reproduce the underlying cause of death (for example, “senility”). Besides, concerning primary health care coverage, we only measure FHS coverage, which is the principal strategy for organizing this level of care in Brazil, but not the only one.

The knowledge that factors of socioeconomic origin influence mortality from oral cancer is well established in the literature. The results of this study indicate that the availability of this knowledge was not sufficient to reduce the inequities related to this outcome in Brazil: the less developed regions had the greatest APC of mortality due to OC and OPC. This study innovates in addressing the IGRs as a population aggregate, which tends to encompass the path of patients with cancer in the health care network. It was possible to identify that mortality rates are decreasing in regions that spent more at the outpatient and hospital levels, which suggests that the investment in health care network is effective for this outcome. Finally, when comparing trends in mortality from oral cancer and oropharyngeal cancer, this study found no evidence that, in Brazil, HPV plays the leading etiological factor in oropharyngeal cancer. However, this conclusion requires studies that measure the virus (and other signs of its role as an etiological factor) in these tumors and the incidence of these diseases.

## Supplementary Information

Supplementary Information.
