# Supplementary material for: The impact of inequalities and health expenditure on mortality due to oral and oropharyngeal cancer in Brazil
Source: Sci Rep. 2021 Jun 18;11:12845. doi: 10.1038/s41598-021-92207-x (PMC8213849; doi:10.1038/s41598-021-92207-x)
Supplement: Supplementary file 1 — Supplementary Information. [file 41598_2021_92207_MOESM1_ESM.docx]

The impact of inequalities and health expenditure on mortality due to oral and oropharyngeal cancer in Brazil - **Supplement**

Amanda Ramos da Cunha^a*^

Alessandro Bigoni^b^

José Leopoldo Ferreira Antunes^c^

Fernando Neves Hugo^d^

^a^Faculty of Dentistry, Federal University of Rio Grande do Sul, 2492 Ramiro Barcelos St, Porto Alegre-RS, 90035-003, Brazil. E-mail: amandaracunha@yahoo.com.br. ^*^Corresponding Author.

^b^School of Public Health, University of São Paulo, 715 Doutor Arnaldo Ave, São Paulo-SP, 01246-904, Brazil. E-mail: alebigoni@usp.br

^c^School of Public Health, University of São Paulo, 715 Doutor Arnaldo Ave, São Paulo-SP, 01246-904, Brazil. E-mail: leopoldo@usp.br

^d^Faculty of Dentistry, Federal University of Rio Grande do Sul, 2492 Ramiro Barcelos St, Porto Alegre-RS, 90035-003, Brazil. E-mail: fernando.hugo@ufrgs.br

Supplementary Chart S1. International Classification of Diseases, tenth revision (ICD-10) codes included in the categories of “oral cancer” and “oropharyngeal cancer.”

| Oral Cancer | ICD-10 |
| --- | --- |
| Malignant neoplasm of dorsal surface of tongue | C02.0 |
| Malignant neoplasm of border of tongue | C02.1 |
| Malignant neoplasm of ventral surface of tongue | C02.2 |
| Malignant neoplasm of anterior two-thirds of tongue, part unspecified | C02.3 |
| Malignant neoplasm of overlapping lesion of tongue | C02.8 |
| Malignant neoplasm of tongue, unspecified | C02.9 |
| Malignant neoplasm of upper gum | C03.0 |
| Malignant neoplasm of lower gum | C03.1 |
| Malignant neoplasm of gum, unspecified | C03.9 |
| Malignant neoplasm of anterior floor of mouth | C04.0 |
| Malignant neoplasm of lateral floor of mouth | C04.1 |
| Malignant neoplasm of overlapping lesion of floor of mouth | C04.8 |
| Malignant neoplasm of floor of mouth, unspecified | C04.9 |
| Malignant neoplasm of hard palate | C05.0 |
| Malignant neoplasm of soft palate | C05.1 |
| Malignant neoplasm of uvula | C05.2 |
| Malignant neoplasm of overlapping lesion of palate | C05.8 |
| Malignant neoplasm of palate, unspecified | C05.9 |
| Malignant neoplasm of cheek mucosa | C06.0 |
| Malignant neoplasm of vestibule of mouth | C06.1 |
| Neoplasia maligna of retromolar area | C06.2 |
| Malignant neoplasm of overlapping lesion of other and unspecified parts of mouth | C06.8 |
| Malignant neoplasm of mouth, unspecified | C06.9 |
| Oropharyngeal Cancer | ICD-10 |
| Malignant neoplasm of base of tongue | C01 |
| Malignant neoplasm of lingual tonsil | C02.4 |
| Malignant neoplasm of tonsillar fossa | C09.0 |
| Malignant neoplasm of tonsillar pillar (anterior) (posterior) | C09.1 |
| Malignant neoplasm of overlapping lesion of tonsil | C09.8 |
| Malignant neoplasm of tonsil, unspecified | C09.9 |
| Malignant neoplasm of vallecula | C10.0 |
| Malignant neoplasm of anterior surface of epiglottis | C10.1 |
| Malignant neoplasm of lateral wall of oropharynx | C10.2 |
| Malignant neoplasm of posterior wall of oropharynx | C10.3 |
| Malignant neoplasm of branchial cleft | C10.4 |
| Malignant neoplasm of overlapping lesion of oropharynx | C10.8 |
| Malignant neoplasm of oropharynx, unspecified | C10.9 |
| Malignant neoplasm of Waldeyer ring | C14.2 |

**Redistribution of ill-defined deaths**

Deaths by ill-defined and unspecified causes were redistributed proportionally, according to the method of reclassifying proposed by the Global Burden of Disease Study 2010 [1]. This technique adopts the terminology "garbage codes" – deaths assigned to codes that should be redistributed to enhance the validity of public health analysis [2] – and this concept covers codes from several chapters of the ICD-10. The GBD Study indicates which groups of garbage codes should be redistributed (and in what proportion) for each underlying cause of death studied. Considering the application only for cancer, the method can be summarized in the following formula, as proposed by Bigoni et al [3].:

$$NDc+\sum{NDg}_{i}*C_{i}$$

Where:

- *NDc* is the number of deaths by oral cancer or oropharyngeal (certified)
- *i* is the garbage code group
- *NDgi* is the number of deaths by a garbage code group
- *C* is the coefficient, which indicates the proportion of the group that must be redistributed to cancer

The garbage code groups and the coefficients proposed by GBD 2010 to correct deaths from oral and other pharyngeal cancer are presented below (Supplementary Chart S2). We adapted this method because we did not consider the same ICD-10 codes used by the GBD 2010 to categorize oral and oropharyngeal cancer. Unlike what was proposed by our study, the GBD considers salivary gland neoplasms as oral cancer and sets hypopharyngeal neoplasms in the same category of oropharynx cancer. For this reason, we deducted the proportion of cases of these anatomical sites from the coefficient of the respective categories.

References:

[1] Lozano, R. *et al*. Global and regional mortality from 235 causes of death for 20 age groups in 1990 and 2010: a systematic analysis for the Global Burden of Disease Study 2010. *Lancet*. **380,** 2095-2128 (2012).

[2] Naghavi, M., Makela, S., Foreman, K., O’Brien, J., Pourmalek, F., Lozano, R. Algorithms for enhancing public health utility of national causes-of-death data. *Popul Health Metr*. **8**, 1–14 (2010).

[3] Bigoni A., Cunha A. R., Antunes J. L. F. Redistributing deaths by ill-defined and unspecified causes on cancer mortality in Brazil. *Rev Saude Publica.* (2021).

Supplementary Chart S2. Global Burden of Disease (GBD) method of ill-defined death redistribution: garbage codes, their respective ICD-10 codes, and coefficients to redistribution.

| Garbage Code | ICD-10 | Mouth cancer | Cancer of other part of pharynx and oropharynx |
| --- | --- | --- | --- |
| All disorders of electrolyte & fluid balance | E86 E870 E871 E872 E873 E874 E875 E876 E877 E878 | 0.06 | 0.04 |
| Cardiogenic shock and other shock | R57 R570 R571 R572 R578 R579 | 0.01 | 0 |
| DIC, cardiac arrest, acute respiratory failure, and coma | D65 I46 I460 I461 I469 J96 J960 J969 R402 | 0.18 | 0.1 |
| Different paralytic syndrome and palsy syndrome | G80 G800 G801 G802 G803 G804 G808 G809 G81 G810 G811 G819 G82 G820 G821 G822 G823 G824 G825 G83 G830 G831 G832 G833 G834 G835 G838 G839 | 0.07 | 0.05 |
| Embolism & thrombosis | I74 I740 I741 I742 I743 I744 I745 I748 I749 | 0.04 | 0.03 |
| Fever, malaise, febrile and convulsions of unknown origin | R50 R508 R509 R53 R56 R560 R568 | 0.09 | 0.05 |
| Ill-defined codes from A00-B99 | A01 A31 A310 A311 A318 A319 A42 A420 A421 A422 A428 A429 A43 A430 A431 A438 A439 A44 A440 A441 A448 A449 A48 A480 A483 A488 A49 A490 A491 A492 A493 A498 A499 A59 A590 A598 A599 A64 A71 A710 A711 A719 A740 A99 B07 B08 B080 B081 B082 B083 B084 B085 B088 B09 B30 B300 B301 B302 B303 B308 B309 B34 B340 B341 B342 B343 B344 B348 B349 B35 B350 B351 B352 B353 B354 B355 B356 B358 B359 B36 B360 B361 B362 B363 B368 B369 B37 B370 B371 B372 B373 B374 B375 B376 B378 B379 B38 B380 B381 B382 B383 B384 B387 B388 B389 B39 B390 B391 B392 B393 B394 B395 B399 B40 B400 B401 B402 B403 B407 B408 B409 B41 B410 B417 B418 B419 B42 B420 B421 B427 B428 B429 B43 B430 B431 B432 B438 B439 B44 B440 B441 B442 B447 B448 B449 B45 B450 B451 B452 B453 B457 B458 B459 B46 B460 B461 B462 B463 B464 B465 B468 B469 B49 B54 B55 B551 B552 B559 B58 B580 B581 B582 B583 B588 B589 B59 B68 B680 B681 B689 B73 B74 B740 B741 B742 B76 B760 B761 B768 B769 B78 B780 B781 B787 B789 B79 B80 B81 B810 B811 B812 B813 B814 B818 B839 B85 B850 B851 B852 B853 B854 B87 B870 B871 B872 B873 B874 B878 B879 B88 B880 B881 B882 B883 B888 B889 B956 B957 B958 B96 B960 B961 B962 B963 B964 B965 B966 B967 B968 B97 B970 B971 B972 B973 B977 B978 | 0.15 | 0 |
| Ill-defined codes from D10-D36.9 | D10 D109 D13 D139 D14 D15 D150 D151 D152 D157 D159 D16 D160 D161 D162 D163 D164 D165 D166 D167 D168 D169 D17 D170 D171 D172 D173 D174 D175 D176 D177 D179 D18 D180 D181 D19 D190 D191 D197 D199 D20 D200 D201 D21 D210 D211 D212 D213 D214 D215 D216 D219 D28 D289 D29 D299 D30 D309 D360 D369 | 0.26 | 0.12 |
| Ill-defined codes from F32-F99 | F32 F320 F321 F322 F323 F328 F329 F33 F330 F331 F332 F333 F334 F338 F339 F34 F340 F341 F348 F349 F38 F380 F381 F388 F39 F40 F400 F401 F402 F408 F409 F41 F410 F411 F412 F413 F418 F419 F42 F420 F421 F422 F428 F429 F43 F430 F431 F432 F438 F439 F44 F440 F441 F442 F443 F444 F445 F446 F447 F448 F449 F45 F450 F451 F452 F453 F454 F458 F459 F48 F480 F481 F488 F489 F50 F508 F509 F51 F510 F511 F512 F513 F514 F515 F518 F519 F52 F520 F521 F522 F523 F524 F525 F526 F527 F528 F529 F53 F530 F531 F538 F539 F54 F55 F59 F60 F600 F601 F602 F603 F604 F605 F606 F607 F608 F609 F61 F62 F620 F621 F628 F629 F63 F630 F631 F632 F633 F638 F639 F64 F640 F641 F642 F648 F649 F65 F650 F651 F652 F653 F654 F655 F656 F658 F659 F66 F660 F661 F662 F668 F669 F68 F680 F681 F688 F69 F70 F700 F701 F708 F709 F71 F710 F711 F718 F719 F72 F720 F721 F728 F729 F73 F730 F731 F738 F739 F78 F780 F781 F788 F789 F79 F790 F791 F798 F799 F80 F800 F801 F802 F803 F808 F809 F81 F810 F811 F812 F813 F818 F819 F82 F83 F84 F840 F841 F842 F843 F844 F845 F848 F849 F88 F89 F90 F900 F901 F908 F909 F91 F910 F911 F912 F913 F918 F919 F92 F920 F928 F929 F93 F930 F931 F932 F933 F938 F939 F94 F940 F941 F942 F948 F949 F95 F950 F951 F952 F958 F959 F98 F980 F981 F982 F983 F984 F985 F986 F988 F989 F99 | 0.3 | 0.2 |
| Ill-defined codes from G43-G58.9 | G43 G430 G431 G432 G433 G438 G439 G44 G440 G441 G442 G443 G444 G448 G47 G470 G471 G472 G474 G478 G479 G50 G500 G501 G508 G509 G51 G510 G511 G512 G513 G514 G518 G519 G52 G520 G521 G522 G523 G527 G528 G529 G53 G530 G531 G532 G533 G538 G54 G540 G541 G542 G543 G544 G545 G546 G547 G548 G549 G55 G550 G551 G552 G553 G558 G56 G560 G561 G562 G563 G564 G568 G569 G57 G570 G571 G572 G573 G574 G575 G576 G578 G579 G58 G580 G587 G588 G589 | 0.24 | 0.11 |
| Ill-defined codes from H00-H99 | H00 H000 H001 H01 H010 H011 H018 H019 H02 H020 H021 H022 H023 H024 H025 H026 H027 H028 H029 H03 H030 H031 H038 H04 H040 H041 H042 H043 H044 H045 H046 H048 H049 H05 H052 H053 H054 H055 H058 H059 H06 H060 H061 H062 H063 H10 H100 H101 H102 H103 H104 H105 H108 H109 H11 H110 H111 H112 H113 H114 H118 H119 H13 H130 H131 H132 H133 H138 H15 H150 H151 H158 H159 H16 H160 H161 H162 H163 H164 H168 H169 H17 H170 H171 H178 H179 H18 H180 H181 H182 H183 H184 H185 H186 H187 H188 H189 H19 H190 H191 H192 H193 H198 H20 H200 H201 H202 H208 H209 H21 H210 H211 H212 H213 H214 H215 H218 H219 H22 H220 H221 H228 H25 H250 H251 H252 H258 H259 H26 H260 H261 H262 H263 H264 H268 H269 H27 H270 H271 H278 H279 H28 H280 H281 H282 H288 H30 H300 H301 H302 H308 H309 H31 H310 H311 H312 H313 H314 H318 H319 H32 H320 H328 H33 H330 H331 H332 H333 H334 H335 H34 H340 H341 H342 H348 H349 H35 H350 H351 H352 H353 H354 H355 H356 H357 H358 H359 H36 H360 H368 H40 H400 H401 H402 H403 H404 H405 H406 H408 H409 H42 H420 H428 H43 H430 H431 H432 H433 H438 H439 H44 H440 H441 H442 H443 H444 H445 H446 H447 H448 H449 H45 H450 H451 H458 H46 H47 H470 H471 H472 H473 H474 H475 H476 H477 H48 H480 H481 H488 H49 H490 H491 H492 H493 H494 H498 H499 H50 H500 H501 H502 H503 H504 H505 H506 H508 H509 H51 H510 H511 H512 H518 H519 H52 H520 H521 H522 H523 H524 H525 H526 H527 H53 H530 H531 H532 H533 H534 H535 H536 H538 H539 H54 H540 H541 H542 H543 H544 H545 H546 H547 H55 H57 H570 H571 H578 H579 H58 H580 H581 H588 H59 H590 H598 H599 H60 H600 H601 H602 H603 H604 H605 H608 H609 H61 H610 H611 H612 H613 H618 H619 H62 H620 H621 H622 H623 H624 H628 H65 H650 H651 H652 H653 H654 H659 H66 H660 H661 H662 H663 H664 H669 H67 H670 H671 H678 H68 H680 H681 H69 H690 H698 H699 H71 H72 H720 H721 H722 H728 H729 H73 H730 H731 H738 H739 H74 H740 H741 H742 H743 H744 H748 H749 H75 H750 H758 H80 H800 H801 H802 H808 H809 H81 H810 H811 H812 H813 H814 H818 H819 H82 H83 H830 H831 H832 H833 H838 H839 H90 H900 H901 H902 H903 H904 H905 H906 H907 H908 H91 H910 H911 H912 H913 H918 H919 H92 H920 H921 H922 H93 H930 H931 H932 H933 H938 H939 H94 H940 H948 H95 H950 H951 H958 H959 | 0.17 | 0.06 |
| Ill-defined codes from K00-K14.9 | K00 K000 K001 K002 K003 K004 K005 K006 K007 K008 K009 K01 K010 K011 K02 K020 K021 K022 K023 K024 K028 K029 K03 K030 K031 K032 K033 K034 K035 K036 K037 K038 K039 K04 K040 K041 K042 K043 K044 K045 K046 K047 K048 K049 K05 K050 K051 K052 K053 K054 K055 K056 K06 K060 K061 K062 K068 K069 K07 K070 K071 K072 K073 K074 K075 K076 K078 K079 K08 K080 K081 K082 K083 K088 K089 K09 K090 K091 K092 K098 K099 K10 K100 K101 K102 K103 K108 K109 K11 K110 K111 K112 K113 K114 K115 K116 K117 K118 K119 K12 K120 K121 K122 K13 K130 K131 K132 K133 K134 K135 K136 K137 K14 K140 K141 K142 K143 K144 K145 K146 K148 K149 | 0.19 | 0.07 |
| Ill-defined codes from L01-L98.9 | L20 L200 L208 L209 L21 L210 L211 L218 L219 L22 L23 L230 L231 L232 L233 L234 L235 L236 L237 L238 L239 L24 L240 L241 L242 L243 L244 L245 L246 L247 L248 L249 L25 L250 L251 L252 L253 L254 L255 L258 L259 L26 L27 L270 L271 L272 L278 L279 L28 L280 L281 L282 L29 L290 L291 L292 L293 L298 L299 L30 L300 L301 L302 L303 L304 L305 L308 L309 L40 L400 L401 L402 L403 L404 L405 L408 L409 L41 L410 L411 L412 L413 L414 L415 L418 L419 L42 L43 L430 L431 L432 L433 L438 L439 L44 L440 L441 L442 L443 L444 L448 L449 L45 L50 L500 L501 L502 L503 L504 L505 L506 L508 L509 L52 L53 L530 L531 L532 L533 L538 L539 L54 L540 L548 L56 L560 L561 L562 L564 L57 L570 L571 L572 L573 L574 L575 L578 L579 L59 L590 L598 L599 L60 L600 L601 L602 L603 L604 L605 L608 L609 L62 L620 L628 L63 L630 L631 L632 L638 L639 L64 L640 L648 L649 L65 L650 L651 L652 L658 L659 L66 L660 L661 L662 L663 L664 L668 L669 L67 L670 L671 L678 L679 L68 L680 L681 L682 L683 L688 L689 L70 L700 L701 L702 L703 L704 L705 L708 L709 L71 L710 L711 L718 L719 L72 L720 L721 L722 L728 L729 L73 L730 L731 L732 L738 L739 L74 L740 L741 L742 L743 L744 L748 L749 L75 L750 L751 L752 L758 L759 L80 L81 L810 L811 L812 L813 L814 L815 L816 L817 L818 L819 L82 L83 L84 L85 L850 L851 L852 L853 L858 L859 L86 L87 L870 L871 L872 L878 L879 L90 L900 L901 L902 L903 L904 L905 L906 L908 L909 L91 L910 L918 L919 L92 L920 L921 L922 L923 L928 L929 L94 L940 L941 L942 L943 L944 L945 L946 L948 L949 L95 L950 L951 L958 L959 L985 L986 L988 L989 | 0.22 | 0.1 |
| Ill-defined codes from M09-M99 | M10 M100 M101 M102 M103 M104 M109 M11 M110 M111 M112 M118 M119 M12 M120 M122 M123 M124 M125 M128 M13 M130 M131 M138 M139 M14 M140 M141 M142 M143 M144 M145 M146 M148 M15 M150 M151 M152 M153 M154 M158 M159 M16 M160 M161 M162 M163 M164 M165 M166 M167 M169 M17 M170 M171 M172 M173 M174 M175 M179 M18 M180 M181 M182 M183 M184 M185 M189 M19 M190 M191 M192 M198 M199 M20 M200 M201 M202 M203 M204 M205 M206 M21 M210 M211 M212 M213 M214 M215 M216 M217 M218 M219 M22 M220 M221 M222 M223 M224 M228 M229 M23 M230 M231 M232 M233 M234 M235 M236 M238 M239 M24 M240 M241 M242 M243 M244 M245 M246 M247 M248 M249 M25 M250 M251 M252 M253 M254 M255 M256 M257 M258 M259 M432 M433 M434 M435 M436 M438 M439 M45 M46 M460 M461 M462 M463 M464 M465 M468 M469 M47 M470 M471 M472 M478 M479 M48 M480 M481 M482 M483 M484 M485 M488 M489 M49 M492 M493 M494 M495 M498 M50 M500 M501 M502 M503 M508 M509 M51 M510 M511 M512 M513 M514 M518 M519 M53 M530 M531 M532 M533 M538 M539 M54 M540 M541 M542 M543 M544 M545 M546 M548 M549 M60 M600 M601 M602 M608 M609 M61 M610 M611 M612 M613 M614 M615 M619 M62 M620 M621 M622 M623 M624 M625 M626 M628 M629 M63 M630 M631 M632 M633 M638 M651 M652 M653 M654 M658 M659 M66 M660 M661 M662 M663 M664 M665 M67 M670 M671 M672 M673 M674 M678 M679 M68 M680 M688 M70 M700 M701 M702 M703 M704 M705 M706 M707 M708 M709 M71 M712 M713 M714 M715 M718 M719 M72 M720 M721 M722 M724 M728 M729 M73 M738 M75 M750 M751 M752 M753 M754 M755 M758 M759 M76 M760 M761 M762 M763 M764 M765 M766 M767 M768 M769 M77 M770 M771 M772 M773 M774 M775 M778 M779 M79 M790 M791 M792 M793 M794 M795 M796 M797 M798 M799 M83 M830 M831 M832 M833 M834 M835 M838 M839 M84 M840 M841 M842 M843 M844 M848 M849 M85 M850 M851 M852 M853 M854 M855 M856 M858 M859 M86 M860 M861 M862 M865 M866 M868 M869 M872 M873 M878 M879 M891 M892 M893 M894 M90 M900 M901 M902 M903 M904 M905 M906 M907 M908 M91 M910 M911 M912 M913 M918 M919 M92 M920 M921 M922 M923 M924 M925 M926 M927 M928 M929 M93 M930 M931 M932 M938 M939 M94 M940 M941 M942 M943 M948 M949 M95 M950 M951 M952 M953 M954 M955 M958 M959 M96 M960 M961 M962 M963 M964 M965 M966 M968 M969 M99 | 0.22 | 0.08 |
| Ill-defined codes from N39.3-N97.8 | N393 N394 N398 N399 N40 N42 N420 N421 N422 N428 N429 N43 N430 N431 N432 N433 N434 N46 N47 N48 N480 N481 N482 N483 N484 N485 N486 N488 N489 N50 N500 N501 N508 N509 N51 N510 N511 N512 N518 N61 N62 N63 N64 N640 N641 N642 N643 N644 N645 N648 N649 N70 N700 N701 N709 N71 N710 N711 N719 N73 N730 N731 N732 N733 N734 N735 N736 N738 N739 N74 N740 N742 N743 N744 N748 N82 N820 N821 N822 N823 N824 N825 N828 N829 N84 N842 N843 N848 N849 N85 N850 N851 N852 N853 N854 N855 N856 N857 N858 N859 N86 N88 N880 N881 N882 N883 N884 N888 N889 N89 N890 N891 N892 N893 N894 N895 N896 N897 N898 N899 N90 N900 N901 N902 N903 N904 N905 N906 N907 N908 N909 N91 N910 N911 N912 N913 N914 N915 N92 N920 N921 N922 N923 N924 N925 N926 N93 N930 N938 N939 N94 N940 N941 N942 N943 N944 N945 N946 N948 N949 N95 N950 N951 N952 N953 N958 N959 N97 N970 N971 N972 N973 N974 N978 | 0.23 | 0.11 |
| Ill-defined codes from Q10-Q84.9 | Q10 Q100 Q101 Q102 Q103 Q360 Q361 Q369 | 0.04 | 0.02 |
| Ill-defined codes from R00-R99 | R012 R02 R03 R030 R031 R04 R040 R041 R042 R048 R049 R07 R070 R071 R072 R073 R074 R10 R100 R101 R102 R103 R104 R11 R12 R13 R14 R15 R16 R160 R161 R162 R17 R18 R19 R190 R191 R192 R193 R194 R195 R196 R198 R20 R200 R201 R202 R203 R208 R21 R22 R220 R221 R222 R223 R224 R227 R229 R23 R230 R231 R232 R233 R234 R238 R25 R250 R251 R252 R253 R258 R26 R260 R261 R262 R268 R27 R270 R278 R29 R290 R291 R292 R293 R294 R296 R298 R30 R300 R301 R309 R31 R32 R33 R34 R35 R36 R39 R390 R391 R392 R398 R40 R400 R401 R41 R410 R411 R412 R413 R418 R42 R43 R430 R431 R432 R438 R44 R440 R441 R442 R443 R448 R45 R450 R451 R452 R453 R454 R455 R456 R457 R458 R46 R460 R461 R462 R463 R464 R465 R466 R467 R468 R47 R470 R471 R478 R48 R480 R481 R482 R488 R49 R490 R491 R492 R498 R51 R52 R520 R521 R522 R529 R55 R58 R59 R590 R591 R599 R60 R600 R601 R609 R61 R610 R611 R619 R62 R620 R628 R629 R63 R630 R631 R632 R633 R634 R635 R638 R68 R680 R681 R682 R683 R688 R69 R70 R700 R701 R71 R72 R73 R730 R739 R74 R740 R748 R749 R75 R76 R760 R761 R762 R768 R769 R77 R770 R771 R772 R778 R779 R78 R786 R787 R788 R789 R79 R790 R798 R799 R80 R81 R82 R820 R821 R822 R823 R824 R825 R826 R827 R828 R829 R83 R830 R831 R832 R833 R834 R835 R836 R837 R838 R839 R84 R840 R841 R842 R843 R844 R845 R846 R847 R848 R849 R85 R850 R851 R852 R853 R854 R855 R856 R857 R858 R859 R86 R860 R861 R862 R863 R864 R865 R866 R867 R868 R869 R87 R870 R871 R872 R873 R874 R875 R876 R877 R878 R879 R89 R890 R891 R892 R893 R894 R895 R896 R897 R898 R899 R90 R900 R908 R91 R92 R93 R930 R931 R932 R933 R934 R935 R936 R937 R938 R94 R940 R941 R942 R943 R944 R945 R946 R947 R948 R96 R960 R961 R98 R99 | 0.32 | 0.16 |
| Ill-defined codes from Z00-Z99 | Z00 Z000 Z001 Z002 Z003 Z004 Z005 Z006 Z008 Z01 Z010 Z011 Z012 Z013 Z014 Z015 Z016 Z017 Z018 Z019 Z02 Z020 Z021 Z022 Z023 Z024 Z025 Z026 Z027 Z028 Z029 Z03 Z030 Z031 Z032 Z033 Z034 Z035 Z036 Z038 Z039 Z04 Z040 Z041 Z042 Z043 Z044 Z045 Z046 Z048 Z049 Z08 Z080 Z081 Z082 Z087 Z088 Z089 Z09 Z090 Z091 Z092 Z093 Z094 Z097 Z098 Z099 Z10 Z100 Z101 Z102 Z103 Z108 Z11 Z110 Z111 Z112 Z113 Z114 Z115 Z116 Z118 Z119 Z12 Z120 Z121 Z122 Z123 Z124 Z125 Z126 Z128 Z129 Z13 Z130 Z131 Z132 Z133 Z134 Z135 Z136 Z137 Z138 Z139 Z20 Z200 Z201 Z202 Z203 Z204 Z205 Z206 Z207 Z208 Z209 Z21 Z22 Z220 Z221 Z222 Z223 Z224 Z225 Z226 Z228 Z229 Z23 Z230 Z231 Z232 Z233 Z234 Z235 Z236 Z237 Z238 Z24 Z240 Z241 Z242 Z243 Z244 Z245 Z246 Z25 Z250 Z251 Z258 Z26 Z260 Z268 Z269 Z27 Z270 Z271 Z272 Z273 Z274 Z278 Z279 Z28 Z280 Z281 Z282 Z288 Z289 Z29 Z290 Z291 Z292 Z298 Z299 Z30 Z300 Z301 Z302 Z303 Z304 Z305 Z308 Z309 Z31 Z310 Z311 Z312 Z313 Z314 Z315 Z316 Z318 Z319 Z32 Z320 Z321 Z33 Z34 Z340 Z348 Z349 Z35 Z350 Z351 Z352 Z353 Z354 Z355 Z356 Z357 Z358 Z359 Z36 Z360 Z361 Z362 Z363 Z364 Z365 Z368 Z369 Z37 Z370 Z371 Z372 Z373 Z374 Z375 Z376 Z377 Z379 Z38 Z380 Z381 Z382 Z383 Z384 Z385 Z386 Z387 Z388 Z39 Z390 Z391 Z392 Z40 Z400 Z408 Z409 Z41 Z410 Z411 Z412 Z413 Z418 Z419 Z42 Z420 Z421 Z422 Z423 Z424 Z428 Z429 Z43 Z430 Z431 Z432 Z433 Z434 Z435 Z436 Z437 Z438 Z439 Z44 Z440 Z441 Z442 Z443 Z448 Z449 Z45 Z450 Z451 Z452 Z453 Z458 Z459 Z46 Z460 Z461 Z462 Z463 Z464 Z465 Z466 Z467 Z468 Z469 Z47 Z470 Z478 Z479 Z48 Z480 Z488 Z489 Z49 Z490 Z491 Z492 Z50 Z500 Z501 Z502 Z503 Z504 Z505 Z506 Z507 Z508 Z509 Z51 Z510 Z511 Z512 Z513 Z514 Z515 Z516 Z518 Z519 Z52 Z520 Z521 Z522 Z523 Z524 Z525 Z526 Z527 Z528 Z529 Z53 Z530 Z531 Z532 Z538 Z539 Z54 Z540 Z541 Z542 Z543 Z544 Z547 Z548 Z549 Z55 Z550 Z551 Z552 Z553 Z554 Z558 Z559 Z56 Z560 Z561 Z562 Z563 Z564 Z565 Z566 Z567 Z57 Z570 Z571 Z572 Z573 Z574 Z575 Z576 Z577 Z578 Z579 Z58 Z580 Z581 Z582 Z583 Z584 Z585 Z586 Z587 Z588 Z589 Z59 Z590 Z591 Z592 Z593 Z594 Z595 Z596 Z597 Z598 Z599 Z60 Z600 Z601 Z602 Z603 Z604 Z605 Z608 Z609 Z61 Z610 Z611 Z612 Z613 Z614 Z615 Z616 Z617 Z618 Z619 Z62 Z620 Z621 Z622 Z623 Z624 Z625 Z626 Z628 Z629 Z63 Z630 Z631 Z632 Z633 Z634 Z635 Z636 Z637 Z638 Z639 Z64 Z640 Z641 Z642 Z643 Z644 Z65 Z650 Z651 Z652 Z653 Z654 Z655 Z658 Z659 Z70 Z700 Z701 Z702 Z703 Z708 Z709 Z71 Z710 Z711 Z712 Z713 Z714 Z715 Z716 Z717 Z718 Z719 Z72 Z720 Z721 Z722 Z723 Z724 Z725 Z726 Z728 Z729 Z73 Z730 Z731 Z732 Z733 Z734 Z735 Z736 Z738 Z739 Z74 Z740 Z741 Z742 Z743 Z748 Z749 Z75 Z750 Z751 Z752 Z753 Z754 Z755 Z758 Z759 Z76 Z760 Z761 Z762 Z763 Z764 Z765 Z768 Z769 Z80 Z800 Z801 Z802 Z803 Z804 Z805 Z806 Z807 Z808 Z809 Z81 Z810 Z811 Z812 Z813 Z814 Z818 Z82 Z820 Z821 Z822 Z823 Z824 Z825 Z826 Z827 Z828 Z83 Z830 Z831 Z832 Z833 Z834 Z835 Z836 Z837 Z84 Z840 Z841 Z842 Z843 Z848 Z85 Z850 Z851 Z852 Z853 Z854 Z855 Z856 Z857 Z858 Z859 Z86 Z860 Z861 Z862 Z863 Z864 Z865 Z866 Z867 Z87 Z870 Z871 Z872 Z873 Z874 Z875 Z876 Z877 Z878 Z88 Z880 Z881 Z882 Z883 Z884 Z885 Z886 Z887 Z888 Z889 Z89 Z890 Z891 Z892 Z893 Z894 Z895 Z896 Z897 Z898 Z899 Z90 Z900 Z901 Z902 Z903 Z904 Z905 Z906 Z907 Z908 Z91 Z910 Z911 Z912 Z913 Z914 Z915 Z916 Z918 Z92 Z920 Z921 Z922 Z923 Z924 Z925 Z926 Z928 Z929 Z93 Z930 Z931 Z932 Z933 Z934 Z935 Z936 Z938 Z939 Z94 Z940 Z941 Z942 Z943 Z944 Z945 Z946 Z947 Z948 Z949 Z95 Z950 Z951 Z952 Z953 Z954 Z955 Z958 Z959 Z96 Z960 Z961 Z962 Z963 Z964 Z965 Z966 Z967 Z968 Z969 Z97 Z970 Z971 Z972 Z973 Z974 Z975 Z978 Z98 Z980 Z981 Z982 Z988 Z99 Z990 Z991 Z992 Z993 Z998 Z999 | 0.34 | 0.21 |
| R54, Senility | R54 | 0.27 | 0.04 |
| Unspecified liver disease | K704 K709 K71 K710 K711 K712 K713 K714 K715 K716 K718 K719 K72 K720 K721 K729 K73 K730 K731 K732 K738 K739 K75 K750 | 0.01 | 0.01 |
| All unspecified site in situ carcinoma and unspecified site Neoplasm of uncertain or unknown behavior | D000 D01 D014 D015 D017 D019 D02 D024 D07 D073 D076 D09 D091 D097 D099 D10 D370 D376 D377 D379 D38 D386 D39 D390 D397 D399 D40 D409 D41 D419 D44 D449 D48 D487 D489 | 14.41 | 4.86 |
| R64, Cachexia | R64 | 1 | 0.44 |
| C14.1-.9, Other and ill- defined sites in the lip, oral cavity and pharynx | C14 C140 C148 | 52.4 | 37.79 |
| C76.0-.9, Malignant neoplasm of other and ill- defined sites | C76 C760 C761 C762 C763 C764 C765 C767 C768 | 6.17 | 5.98 |
| C80.0-.9, Malignant neoplasm without specification of site | C80 C800 C809 | 0 | 3.09 |

Supplementary Chart S3. Exposure variables: year, data sources, and calculation.

| Variable | Year | Data Source | Calculation |
| --- | --- | --- | --- |
| Human Development Index | 2010 | United Nations Development Program | HDI was collected by city. For IGR, we calculated a weighted average with the number of inhabitants in 2018 in each town of the IGR as the weighting factor. |
| Family Health Strategy coverage | 2018 | Information System on Primary Health Care | The number of FHS was collected by city for each 2018’s month. We divided this total by the city's number of inhabitants in 2018, multiplied by 3000*, and multiplied by 100 to obtain the percentual coverage for each town by month. The annual coverage is the mean of the monthly coverage. For IGR, we calculated a weighted average with the number of inhabitants in 2018 in each town of the IGR as the weighting factor. |
| Government expenditure – Outpatient | 2018 | Ambulatory Information System (SIA/SUS) | The expenditure amount in each IGR was collected in Brazilian Reals. We divided this total by the inhabitants' number of the IGR in 2018 to obtain the *per capita* amount. |
| Government expenditure – Hospital | 2018 | Hospital Information System (SIH/SUS) |  |

*According to the Brazil’s Ministry of Health, “3,000” is the ideal number of inhabitants for each FHT. Source: Brazil. Ministry of Health. Gabinete do Ministro. Portaria Nº 2355, de 10 de outubro de 2013.

Supplementary Table S1. North region: Annual percent change (APC), 95% confidence interval (IC_95%_), and trends of oral (OC) and oropharyngeal (OPC) death rates, by intermediate geographic regions. Brazil, 1996-2018.

| Intermediate Region | State | OC-APC(IC_95%_) | OC-Trend | OPC-APC(IC_95%_) | OPH-Trend |
| --- | --- | --- | --- | --- | --- |
| Altamira | Pará | 1.88(-2.02;5.93) | ↔ | 0.68(-3.53;5.07) | ↔ |
| Araguaína | Tocantins | 1.14(-1.08;3.41) | ↔ | 0.42(-2.99;3.96) | ↔ |
| Belém | Pará | -0.11(-1.53;1.33) | ↔ | -0.48(-1.95;1.02) | ↔ |
| Boa Vista | Roraima | -1.00(-4.10;2.20) | ↔ | -3.38(-8.03;1.51) | ↔ |
| Breves | Pará | 3.43(-0.12;7.10) | ↔ | 5.18(0.42;10.17) | ↑ |
| Castanhal | Pará | 1.12(0.11;2.14) | ↑ | 2.91(1.36;4.48) | ↑ |
| Cruzeiro do Sul | Acre | 0.03(-4.87;5.19) | ↔ | -0.75(-5.31;4.04) | ↔ |
| Gurupi | Tocantins | 2.70(0.05;5.41) | ↔ | 1.78(-4.94;8.99) | ↔ |
| Ji-Paraná | Rondônia | -0.33(-2.48;1.86) | ↔ | -1.22(-3.81;1.44) | ↔ |
| Lábrea | Amazonas | 4.71(-0.38;10.05) | ↔ | 7.33(2.56;12.32) | ↑ |
| Macapá | Amapá | 1.42(-1.65;4.58) | ↔ | -1.01(-5.16;3.32) | ↔ |
| Manaus | Amazonas | -0.91(-2.03;0.21) | ↔ | 0.79(-0.79;2.39) | ↔ |
| Marabá | Pará | 0.29(-2.63;3.30) | ↔ | 1.57(0.60;2.56) | ↑ |
| Oiapoque - Porto Grande | Amapá | 4.47(-4.91;14.77) | ↔ | 1.33(-9.61;13.58) | ↔ |
| Palmas | Tocantins | 1.96(-1.67;5.73) | ↔ | 1.21(-3.38;6.03) | ↔ |
| Parintins | Amazonas | 1.76(-0.91;4.51) | ↔ | 4.06(1.05;7.15) | ↑ |
| Porto Velho | Rondônia | -1.31(-3.70;1.15) | ↔ | 1.37(-0.67;3.44) | ↔ |
| Redenção | Pará | 0.62(-2.62;3.98) | ↔ | 0.94(-3.41;5.49) | ↔ |
| Rio Branco | Acre | -1.85(-4.41;0.78) | ↔ | -2.40(-4.45;-0.30) | ↓ |
| Rorainópolis - Caracaraí | Roraima | -1.23(-9.51;7.80) | ↔ | 5.73(-6.27;19.26) | ↔ |
| Santarém | Pará | 0.41(-2.17;3.06) | ↔ | 0.58(-2.94;4.24) | ↔ |
| Tefé | Amazonas | 1.06(-2.65;4.92) | ↔ | 3.90(-0.02;7.98) | ↔ |

↑ Increasing trend; ↓ Decreasing trend; ↔ Stationary trend

Supplementary Table S2. Northeast region: Annual percent change (APC), 95% confidence interval (IC_95%_), and trends of oral (OC) and oropharyngeal (OPC) death rates, by intermediate geographic regions. Brazil, 1996-2018.

| Intermediate Region | State | OC-APC(IC_95%_) | OC-Trend | OPC-APC(IC_95%_) | OPH-Trend |
| --- | --- | --- | --- | --- | --- |
| Aracaju | Sergipe | 1.94(0.49;3.41) | ↑ | -0.56(-3.39;2.36) | ↔ |
| Arapiraca | Alagoas | 2.73(1.48;3.99) | ↑ | 2.88(1.41;4.38) | ↑ |
| Barreiras | Bahia | 2.18(-0.08;4.50) | ↔ | 0.68(-2.60;4.07) | ↔ |
| Caicó | Rio Grande do Norte | 2.59(0.50;4.72) | ↑ | 3.96(1.21;6.78) | ↑ |
| Campina Grande | Paraíba | 3.62(1.18;6.12) | ↑ | 3.10(1.05;5.20) | ↑ |
| Caruaru | Pernambuco | 0.21(-0.86;1.30) | ↔ | 1.85(1.06;2.64) | ↑ |
| Caxias | Maranhão | 3.72(1.84;5.62) | ↑ | 5.89(2.85;9.02) | ↑ |
| Corrente - Bom Jesus | Piauí | -0.83(-6.45;5.14) | ↔ | 5.34(1.77;9.04) | ↑ |
| Crateús | Ceará | 2.38(-0.47;5.31) | ↔ | 1.42(-2.95;5.99) | ↔ |
| Feira de Santana | Bahia | 1.73(0.24;3.26) | ↑ | 3.98(2.38;5.61) | ↑ |
| Floriano | Piauí | 2.87(-0.93;6.81) | ↔ | 2.62(-3.71;9.38) | ↔ |
| Fortaleza | Ceará | -0.75(-1.42;-0.08) | ↓ | 0.32(-0.44;1.09) | ↔ |
| Guanambi | Bahia | 3.57(1.48;5.70) | ↑ | 3.84(1.09;6.66) | ↑ |
| Iguatu | Ceará | -2.56(-4.27;-0.82) | ↓ | -1.78(-5.20;1.76) | ↔ |
| Ilhéus – Itabuna | Bahia | 1.50(0.38;2.64) | ↑ | 3.53(2.29;4.79) | ↑ |
| Imperatriz | Maranhão | 1.73(0.21;3.27) | ↑ | 4.41(2.82;6.03) | ↑ |
| Irecê | Bahia | 3.45(0.57;6.41) | ↑ | 4.35(1.87;6.90) | ↑ |
| Itabaiana | SeIGRpe | 5.43(2.07;8.90) | ↑ | 3.12(-0.83;7.23) | ↔ |
| João Pessoa | Paraíba | 1.20(-1.70;4.19) | ↔ | 1.30(0.27;2.34) | ↑ |
| Juazeiro | Bahia | 4.09(1.76;6.48) | ↑ | 4.59(1.99;7.25) | ↑ |
| Juazeiro do Norte | Ceará | 2.37(0.33;4.46) | ↑ | 3.69(1.92;5.48) | ↑ |
| Maceió | Alagoas | 0.53(-0.86;1.94) | ↔ | 0.88(-0.62;2.42) | ↔ |
| Mossoró | Rio Grande do Norte | 3.30(0.22;6.46) | ↑ | 3.92(2.65;5.19) | ↑ |
| Natal | Rio Grande do Norte | 1.39(0.21;2.59) | ↑ | 1.94(0.83;3.06) | ↑ |
| Parnaíba | Piauí | 2.71(0.07;5.43) | ↑ | 2.79(-0.29;5.97) | ↔ |
| Patos | Paraíba | 2.53(0.57;4.52) | ↑ | 2.41(-0.69;5.60) | ↔ |
| Paulo Afonso | Bahia | 2.30(1.15;3.46) | ↑ | 4.85(3.45;6.27) | ↑ |
| Petrolina | Pernambuco | 1.14(0.06;2.22) | ↑ | 0.62(-1.26;2.54) | ↔ |
| Picos | Piauí | 4.82(1.25;8.50) | ↑ | 1.75(-1.49;5.09) | ↔ |
| Presidente Dutra | Maranhão | 2.74(-0.08;5.63) | ↔ | 5.08(1.39;8.90) | ↑ |
| Quixadá | Ceará | -1.49(-3.57;0.64) | ↔ | 0.96(-1.44;3.43) | ↔ |
| Recife | Pernambuco | -1.29(-1.79;-0.79) | ↓ | 1.69(0.95;2.43) | ↑ |
| Salvador | Bahia | -2.37(-2.79;-1.95) | ↓ | -0.37(-1.19;0.46) | ↔ |
| Santa Inês - Bacabal | Maranhão | 1.57(-1.12;4.34) | ↔ | 2.12(-0.84;5.17) | ↔ |
| Santo Antônio de Jesus | Bahia | 1.62(0.37;2.88) | ↑ | 1.90(0.43;3.39) | ↑ |
| São Luís | Maranhão | 0.69(-0.94;2.34) | ↔ | 1.97(0.66;3.30) | ↑ |
| São Raimundo Nonato | Piauí | 2.42(-2.53;7.62) | ↔ | 8.86(2.24;15.91) | ↑ |
| Serra Talhada | Pernambuco | 2.77(1.28;4.27) | ↑ | -1.20(-3.55;1.21) | ↔ |
| Sobral | Ceará | 0.51(-1.29;2.35) | ↔ | 0.42(-1.69;2.58) | ↔ |
| Sousa - Cajazeiras | Paraíba | 2.13(0.03;4.28) | ↑ | 2.69(-0.87;6.38) | ↔ |
| Teresina | Piauí | 2.07(0.86;3.30) | ↑ | 2.74(0.61;4.91) | ↑ |
| Vitória da Conquista | Bahia | 2.74(1.37;4.14) | ↑ | 3.00(1.37;4.66) | ↑ |

↑ Increasing trend; ↓ Decreasing trend; ↔ Stationary trend

Supplementary Table S3. Southeast region: Annual percent change (APC), 95% confidence interval (IC_95%_), and trends of oral (OC) and oropharyngeal (OPC) death rates, by intermediate geographic regions. Brazil, 1996-2018.

| Intermediate Region | State | OC-APC | OC-Trend | OPC-APC | OPH-Trend |
| --- | --- | --- | --- | --- | --- |
| Araçatuba | São Paulo | -2.62(-4.09;-1.13) | ↓ | -0.64(-1.76;0.48) | ↔ |
| Araraquara | São Paulo | 0.82(-0.84;2.51) | ↔ | -1.51(-2.44;-0.57) | ↓ |
| Barbacena | Minas Gerais | 0.84(-1.29;3.02) | ↔ | 1.72(0.42;3.04) | ↑ |
| Bauru | São Paulo | -1.07(-2.15;0.02) | ↔ | 0.10(-1.20;1.42) | ↔ |
| Belo Horizonte | Minas Gerais | -0.94(-1.64;-0.23) | ↓ | 1.91(1.23;2.61) | ↑ |
| Cachoeiro do Itapemirim | Espírito Santo | 0.43(-1.51;2.40) | ↔ | 0.62(-1.97;3.28) | ↔ |
| Campinas | São Paulo | -1.03(-1.24;-0.81) | ↓ | -1.76(-2.33;-1.18) | ↓ |
| Campos dos Goytacazes | Rio de Janeiro | -0.01(-2.13;2.16) | ↔ | -0.61(-1.72;0.53) | ↔ |
| Colatina | Espírito Santo | 1.29(-0.51;3.12) | ↔ | 3.95(2.47;5.46) | ↑ |
| Divinópolis | Minas Gerais | 1.47(0.00;2.96) | ↑ | 2.05(0.65;3.48) | ↑ |
| Governador Valadares | Minas Gerais | 3.23(1.70;4.79) | ↑ | 3.44(1.58;5.34) | ↑ |
| Ipatinga | Minas Gerais | 1.95(-0.16;4.12) | ↔ | 1.35(-0.36;3.10) | ↔ |
| Juíz de Fora | Minas Gerais | 0.69(-0.06;1.44) | ↔ | -0.05(-1.40;1.33) | ↔ |
| Macaé - Rio das Ostras - Cabo Frio | Rio de Janeiro | -0.58(-1.82;0.67) | ↔ | -3.09(-4.76;-1.40) | ↓ |
| Marília | São Paulo | -0.69(-2.17;0.81) | ↔ | -0.84(-2.05;0.38) | ↔ |
| Montes Claros | Minas Gerais | 2.31(1.06;3.57) | ↑ | 4.12(2.75;5.51) | ↑ |
| Patos de Minas | Minas Gerais | 0.70(-1.87;3.34) | ↔ | 3.05(0.72;5.42) | ↑ |
| Petrópolis | Rio de Janeiro | -0.53(-1.80;0.75) | ↔ | -0.96(-1.99;0.08) | ↔ |
| Pouso Alegre | Minas Gerais | -0.09(-1.25;1.09) | ↔ | -0.24(-1.32;0.85) | ↔ |
| Presidente Prudente | São Paulo | -0.74(-1.59;0.11) | ↔ | -1.11(-2.29;0.07) | ↔ |
| Ribeirão Preto | São Paulo | -0.45(-1.64;0.76) | ↔ | -1.13(-2.30;0.05) | ↔ |
| Rio de Janeiro | Rio de Janeiro | -2.51(-3.20;-1.82) | ↓ | -2.02(-3.01;-1.01) | ↓ |
| São José do Rio Preto | São Paulo | -1.21(-1.90;-0.51) | ↓ | -2.06(-3.40;-0.70) | ↓ |
| São José dos Campos | São Paulo | -0.85(-1.70;0.00) | ↔ | -2.01(-2.75;-1.26) | ↓ |
| São Mateus | Espírito Santo | 2.52(0.32;4.77) | ↑ | 0.97(-2.23;4.27) | ↔ |
| São Paulo | São Paulo | -1.87(-2.14;-1.59) | ↓ | -1.11(-1.73;-0.49) | ↓ |
| Sorocaba | São Paulo | -1.08(-1.62;-0.54) | ↓ | -2.12(-2.67;-1.57) | ↓ |
| Teófilo Otoni | Minas Gerais | 1.37(-1.13;3.93) | ↔ | 3.97(1.91;6.08) | ↑ |
| Uberaba | Minas Gerais | -0.38(-2.22;1.50) | ↔ | 1.90(-0.82;4.71) | ↔ |
| Uberlândia | Minas Gerais | -0.03(-1.09;1.04) | ↔ | -0.01(-1.60;1.61) | ↔ |
| VaIGRnha | Minas Gerais | 0.76(-0.52;2.06) | ↔ | 1.69(0.86;2.52) | ↑ |
| Vitória | Espírito Santo | 0.36(-0.75;1.47) | ↔ | -1.40(-3.12;0.35) | ↔ |
| Volta Redonda - Barra Mansa | Rio de Janeiro | 1.38(-0.46;3.25) | ↔ | -1.00(-3.16;1.20) | ↔ |

↑ Increasing trend; ↓ Decreasing trend; ↔ Stationary trend

Supplementary Table S4. South region: Annual percent change (APC), 95% confidence interval (IC_95%_), and trends of oral (OC) and oropharyngeal (OPC) death rates, by intermediate geographic regions. Brazil, 1996-2018.

| Intermediate Region | State | OC-APC | OC-Trend | OPC-APC | OPH-Trend |
| --- | --- | --- | --- | --- | --- |
| Blumenau | Santa Catarina | -1.33(-2.29;-0.35) | ↓ | -1.62(-2.70;-0.52) | ↓ |
| Caçador | Santa Catarina | 0.21(-3.24;3.78) | ↔ | -0.17(-4.33;4.17) | ↔ |
| Cascavel | Paraná | -1.08(-1.85;-0.30) | ↓ | -0.10(-1.71;1.55) | ↔ |
| Caxias do Sul | Rio Grande do Sul | -0.59(-1.97;0.81) | ↔ | -1.04(-2.62;0.56) | ↔ |
| Chapecó | Santa Catarina | -1.62(-2.78;-0.45) | ↓ | -2.86(-3.92;-1.79) | ↓ |
| Criciúma | Santa Catarina | -0.34(-2.05;1.40) | ↔ | 0.59(-0.88;2.09) | ↔ |
| Curitiba | Paraná | -1.91(-2.86;-0.96) | ↓ | -1.70(-2.53;-0.87) | ↓ |
| Florianópolis | Santa Catarina | -2.39(-3.79;-0.98) | ↓ | 2.14(0.58;3.73) | ↑ |
| Guarapuava | Paraná | 0.67(-0.98;2.34) | ↔ | 1.19(-0.74;3.15) | ↔ |
| Ijuí | Rio Grande do Sul | -1.46(-2.84;-0.07) | ↓ | 0.02(-1.65;1.71) | ↔ |
| Joinville | Santa Catarina | -0.92(-1.96;0.14) | ↔ | -0.83(-2.31;0.68) | ↔ |
| Lages | Santa Catarina | -1.93(-5.02;1.26) | ↔ | 0.79(-2.01;3.66) | ↔ |
| Londrina | Paraná | 0.09(-0.56;0.75) | ↔ | -0.77(-1.42;-0.12) | ↓ |
| Maringá | Paraná | -1.24(-2.18;-0.28) | ↓ | 0.32(-0.46;1.10) | ↔ |
| Passo Fundo | Rio Grande do Sul | -1.26(-2.31;-0.20) | ↓ | -0.45(-1.50;0.61) | ↔ |
| Pelotas | Rio Grande do Sul | -0.71(-2.30;0.90) | ↔ | 2.02(1.04;3.01) | ↑ |
| Ponta Grossa | Paraná | 0.09(-0.71;0.90) | ↔ | -2.29(-3.45;-1.12) | ↓ |
| Porto Alegre | Rio Grande do Sul | -0.63(-1.30;0.04) | ↔ | -0.62(-1.43;0.20) | ↔ |
| Santa Cruz do Sul - Lajeado | Rio Grande do Sul | -2.51(-4.69;-0.29) | ↓ | -0.01(-1.51;1.51) | ↔ |
| Santa Maria | Rio Grande do Sul | -2.36(-3.58;-1.13) | ↓ | -0.17(-1.42;1.08) | ↔ |
| Uruguaiana | Rio Grande do Sul | -0.39(-3.36;2.67) | ↔ | -0.16(-2.34;2.08) | ↔ |

↑ Increasing trend; ↓ Decreasing trend; ↔ Stationary trend

Supplementary Table S5. Midwest region: Annual percent change (APC), 95% confidence interval (IC_95%_), and trends of oral (OC) and oropharyngeal (OPC) death rates, by intermediate geographic regions. Brazil, 1996-2018.

| Intermediate Region | State | OC-APC | OC-Trend | OPC-APC | OPH-Trend |
| --- | --- | --- | --- | --- | --- |
| Barra do Garças | Mato Grosso | 5.31(3.25;7.42) | ↑ | 2.94(-2.12;8.28) | ↔ |
| Cáceres | Mato Grosso | -1.36(-5.05;2.46) | ↔ | 1.08(-4.72;7.23) | ↔ |
| Campo Grande | Mato Grosso do Sul | -0.16(-1.17;0.85) | ↔ | -1.39(-3.03;0.28) | ↔ |
| Corumbá | Mato Grosso do Sul | 0.79(-1.21;2.83) | ↔ | -0.64(-2.58;1.35) | ↔ |
| Cuiabá | Mato Grosso | -0.74(-3.06;1.63) | ↔ | 0.27(-1.12;1.67) | ↔ |
| Distrito Federal | Distrito Federal | -0.84(-2.98;1.36) | ↔ | 0.43(-0.60;1.47) | ↔ |
| Dourados | Mato Grosso do Sul | -1.48(-2.68;-0.27) | ↓ | 0.83(-1.28;2.98) | ↔ |
| Goiânia | Goiás | 0.73(-0.68;2.17) | ↔ | 1.42(0.60;2.24) | ↑ |
| Itumbiara | Goiás | -0.41(-2.91;2.16) | ↔ | 0.14(-1.88;2.20) | ↔ |
| Luziânia - Águas Lindas de Goiás | Goiás | -0.65(-2.83;1.59) | ↔ | 1.00(-0.86;2.89) | ↔ |
| Porangatu - Uruaçu | Goiás | 2.00(0.37;3.65) | ↔ | 0.04(-1.97;2.09) | ↔ |
| Rio Verde | Goiás | -0.58(-3.75;2.69) | ↔ | 0.83(-0.42;2.10) | ↔ |
| Rondonópolis | Mato Grosso | 3.02(-1.04;7.25) | ↔ | 0.94(-1.61;3.57) | ↔ |
| São Luís de Montes Belos - Iporá | Goiás | 5.31(2.53;8.18) | ↑ | 0.15(-3.66;4.12) | ↔ |
| Sinop | Mato Grosso | 0.77(-2.46;4.12) | ↔ | -0.83(-3.23;1.62) | ↔ |

↑ Increasing trend; ↓ Decreasing trend; ↔ Stationary trend
